# AI-2 does not function as a quorum sensing molecule in *Campylobacter jejuni *during exponential growth *in vitro*

**DOI:** 10.1186/1471-2180-9-214

**Published:** 2009-10-08

**Authors:** Kathryn Holmes, Tim J Tavender, Klaus Winzer, Jerry M Wells, Kim R Hardie

**Affiliations:** 1 Pathogens: Molecular Microbiology, BBSRC Institute of Food Research, Norwich Research Park, Colney, Norwich NR4 7UA, UK; 2 School of Molecular Medical Sciences, Centre for Biomolecular Sciences, The University of Nottingham, Clifton Boulevard, University Park, Nottingham NG7 2RD, UK; 3 Department of Clinical Medicine, Trinity College, Adelaide and Meath Hospital, Dublin 24, Ireland; 4 Michael Smith Building, Faculty of Life Sciences, University of Manchester, Manchester M13 9PT, UK; 5 Host-Microbe Interactomics, Animal Sciences Department, University of Wageningen, The Netherlands

## Abstract

**Background:**

*Campylobacter jejuni *contains a homologue of the *luxS *gene shown to be responsible for the production of the signalling molecule autoinducer-2 (AI-2) in *Vibrio harveyi *and *Vibrio cholerae*. The aim of this study was to determine whether AI-2 acted as a diffusible quorum sensing signal controlling *C. jejuni *gene expression when it is produced at high levels during mid exponential growth phase.

**Results:**

AI-2 activity was produced by the parental strain NCTC 11168 when grown in rich Mueller-Hinton broth (MHB) as expected, but interestingly was not present in defined Modified Eagles Medium (MEM-α). Consistent with previous studies, the *luxS *mutant showed comparable growth rates to the parental strain and exhibited decreased motility halos in both MEM-α and MHB. Microarray analysis of genes differentially expressed in wild type and *luxS *mutant strains showed that many effects on mRNA transcript abundance were dependent on the growth medium and linked to metabolic functions including methionine metabolism. Addition of exogenously produced AI-2 to the wild type and the *luxS *mutant, growing exponentially in either MHB or MEM-α did not induce any transcriptional changes as analysed by microarray.

**Conclusion:**

Taken together these results led us to conclude that there is no evidence for the role of AI-2 in cell-to-cell communication in *C. jejuni *strain NCTC 11168 under the growth conditions used, and that the effects of the *luxS *mutation on the transcriptome are related to the consequential loss of function in the activated methyl cycle.

## Background

*Campylobacter jejuni *is the most common cause of food-borne diarrhoeal illness in the developed world. In 2000 there were approximately 60 000 reported cases in England and Wales [1], and there is an estimated 4 million infections (with between 200 and 1000 deaths) each year in the United States [2].

In humans, *Campylobacter *infection causes a range of symptoms from mild, watery diarrhoea to severe, bloody diarrhoea. The illness is self-limiting but infection with certain serotypes is a common antecedent to Guillain-Barré syndrome [3,4]. Reactive arthritis also occurs in approximately 2% of *C. jejuni *enteritis [5,6].

In many species of bacteria including enteric pathogens such as *Escherichia coli*, *Salmonella enterica*, and *Vibrio cholerae*, quorum sensing is thought to play a role in the expression of factors involved in diverse processes such as biofilm formation and pathogenesis [7]. Quorum sensing is the process by which bacteria sense cell density via the synthesis, secretion and detection of signalling molecules commonly known as autoinducers. Whole communities of bacteria are able to control and initiate a concerted response by sensing a threshold concentration of small diffusible signalling molecules when a certain cell density or quorum is reached [8-10].

The only quorum sensing system shared by both Gram-negative and Gram-positive bacteria involves production of autoinducer-2 (AI-2), first discovered as a regulator of bioluminescence in *Vibrio harveyi *[11]. The precursor of AI-2, 4,5-dihydroxy-2,3-pentanedione (DPD), is produced by the enzyme LuxS which has been identified in over 55 different species [10,12]. DPD undergoes cyclisation to form furanone derivatives which possess the ability to induce bioluminescence in *V. harveyi*. Since many bacteria produce the LuxS enzyme, and thus the AI-2 signal, a role of this molecule in inter-species communication has been suggested [12-16]. Different bacteria respond to AI-2 in different ways. Some, notably *Vibrio sp*., detect the presence of AI-2 using specific two component signal transduction to initiate a phospho-relay [17-19]. Others, like *Salmonella *and *Escherichia coli *possess ABC transporter proteins which import and modify AI-2 [16,20-22]. In each of these scenarios, the precise chemical nature of AI-2 appears to differ since the binding protein components have been shown to interact with different, but structurally related molecules. The LuxP AI-2 binding protein of *V. harveyi *was co-crystallized with a furanosyl-borate diester (3A-methyl-5,6-dihydro-furo(2,3-D)(1,3,2)dioxaborole-2,2,6,6A-tetraol; *S*-THMF-borate) [23], whilst LsrB of *S. entericia *serovar Typhimurium was found in complex with (*2R*, 4S)-2-methyl-2,3,3,4-tetrahydroxytetrahydrofuran (*R*-THMF) [24]. Other cyclisation derivatives of DPD such as 4-hydroxy-5-methyl-3(*2H*)-furanone (MHF) or a furanosyl carbonate diester [25] have also been shown to possess AI-2 activity [14,26].

The LuxS enzyme is an established part of the activated methyl cycle (AMC) that generates *S*-adenosyl-L-methionine (SAM) the methyl donor for methylation of RNA, DNA, proteins and certain metabolites. In this cycle, SAM is first converted to *S*-adenosyl-L-homocysteine (SAH) which is then detoxified by the Pfs enzyme to generate adenine and *S*-ribosyl-L-homocyteine (SRH), the substrate of the LuxS enzyme. In the conversion of SRH to homocysteine, DPD is produced as a byproduct and derivatives of this with AI-2 activity are found in culture supernatants [14,26]. The homocysteine moiety is then converted to methionine and subsequently, SAM. Using AI-2 induced bioluminescence of *V. harveyi *as a reporter system, numerous species of bacteria have been shown to produce AI-2 activity including *Helicobacter pylori *[27], *E. coli and Salmonella enterica *serovar Typhimurium [22,28,29], *Neisseria meningitidis *[30-32], *Haemophilus influenza *[33]*Clostridium difficile *[34] and *C. jejuni *[35].

Many of the AI-2 producing bacteria studied are pathogens, and currently numerous reports concluding that LuxS and AI-2 contribute to novel signalling systems exist, although critical evaluation of this data suggests that further studies are required to verify these observations [10,26,36-38]. The potential importance of LuxS in recycling intermediates in the activated methyl cycle via the conversion of SRH to homocysteine and then methionine should not be overlooked. Indeed the disruption of *luxS *itself could decrease the virulence of a pathogen through metabolic perturbations without any involvement of AI-2 in cell-to-cell signalling. Support for this hypothesis comes from two recent studies in *Neisseria meningitidis *where evidence for a proteomic or transcriptional response to AI-2 was lacking [31,32], but the mutant was significantly attenuated *in vivo *[30,39].

Discrimination between the two roles of LuxS/AI-2 is somewhat hazardous. It is complicated by the existence of strain variation of LuxS-dependent phenotypes (e.g. in *Serratia *[40]), and is likely influenced by the immediate environment, i.e. whether it is replete or deficient in nutrients that can repair a metabolic imbalance. To establish a cell-cell communication defect as the underlying cause of an altered phenotype relies on addition of purified signal molecule at an appropriate time and concentration to the cells in the environment under study. Addition of AI-2 or DPD to biofilm communities has revealed that some organisms require low levels (amounts undetectable in the *V. harveyi *bioluminescent assay (0.08 nM DPD) effectively restored phenotypes for oral commensals *Streptococcus oralis *and *Actinomyces reslundii *whilst high levels did not [41]); and others require levels similar to those encountered *in vivo *to complement altered phenotypes exhibited by *luxS *mutants (e.g. in *Staphylococcus epidermidis *[42]). *In vivo *levels of DPD are in the μM range (e.g. 1.95 μM *V. harveyi *and 0.26 μM *Strept. mutans *[43]) Establishing a definitive role for disruption of the AMC in the maintenance of a phenotype may also be problematic. It cannot be predicted that the transcription of all the genes encoding AMC participating enzymes will alter upon interruption of the cycle, as biochemical pathways are often controlled by regulation of one or two key enzymes. Although SAM levels influence methionine *de novo *synthesis in enteric bacteria, AMC disruption may not result in major changes in gene expression as growth media contain all the methionine and SAM required by the cells. An initial step towards greater understanding of the consequences of AI-2 production and *luxS *inactivation would be to study transcriptome changes under conditions where it had been established that AI-2 is produced, and compare this to non-AI-2-containing conditions.

Planktonic, exponentially growing *C. jejuni *has been shown to produce functional AI-2 capable of inducing bioluminescence in a *V. harveyi *bioassay whereas culture supernatants from an isogenic *luxS *mutant strain had no effect on bioluminescence [35]. The *C. jejuni luxS *mutant was comparable to the wild type in its growth rate and its ability to resist oxidative stress and invade Caco-2 monolayers, however it showed significantly decreased motility in semisolid media leading to the suggestion that a quorum sensing role of AI-2 in *C. jejuni *could involve regulation of motility [35]. In line with this, a null mutation of *luxS *in *C. jejuni *strain 81116 reduced motility and transcription of *flaA *[44]. Recently, the effect of *luxS *mutation in *C. jejuni *strain 81-176 on global gene expression has been reported to be limited, with gene expression modulations focused primarily upon genes involved in motility and metabolism [37]. With the aim of gaining further insight into the potential role of AI-2 as a quorum sensing molecule in *C. jejuni *we set out to determine whether the repertoire of gene expression changes influenced by LuxS was strain dependent, and address the primary role of LuxS in metabolism as suggested by He *et al*., 2008 [37] To do this, we analysed the transcriptome of *C. jejuni *NCTC 11168 and its isogenic *luxS *mutant grown in both defined and complex media. Furthermore, exogenous *in vitro*-produced AI-2 was added back to growing cultures of the *luxS *mutant to monitor the transcriptional response induced by this extracellular signal.

## Methods

### *C. jejuni *strains and growth conditions

The bacterial strains used in this study were kindly donated by Simon Park and Karen Elvers (University of Surrey). *C. jejuni *strain NCTC 11168 (wild type) and its isogenic *luxS *mutant, LuxS01 [35] were routinely grown at 42°C under microaerobic conditions (10% CO_2_, 85% N_2_, 5% O_2_; all vol/vol) on Skirrow agar plates, in Mueller Hinton broth (MHB; Oxoid, Basingstoke, UK), or in MEM-α medium (Invitrogen, UK) on an orbital shaker (380 rpm) inside a MACS-MG-1000 controlled atmosphere workstation (Don Whitley Scientific, UK). When required, kanamycin at a final concentration of 25 μg ml^-1 ^(Sigma-Aldrich, UK) was added to the medium. To test for AI-2 activity, 50 ml of MHB or MEM-α was inoculated with *C. jejuni *wild type or *luxS *mutant grown on Skirrow agar and incubated overnight (16-18 h). A 3% inoculum was then used to inoculate a fresh 50 ml broth and grown to late logarithmic phase (approx. 8 h; determined by viable counts and OD_600_). Samples were taken at intervals (typically 8 h) during the logarithmic growth phase to test for AI-2 activity using the *V. harveyi *bioassay. For this assay 1 ml was removed from each culture and centrifuged at 12000 *g*, 4°C for 10 min. The supernatant was then filter-sterilised with a 0.2 μm filter unit (Millipore) and stored at -80°C before analysis.

### Motility Assays

Motility assays were performed as described by Elvers and Park [35] using MHB and MEM-α broth, respectively, both containing 0.4% (wt/vol) agar. Plates were incubated at 37°C and 42°C and motility halos were examined after 16 h, 24 h and 48 h. Parallel experiments were performed on cultures grown in the presence or absence of exogenous AI-2.

### Analysis of culture supernatants for AI-2 activity

Cell-free culture supernatants were prepared by centrifugation and 0.2 μm filtration.

AI-2 activity in supernatants was analysed as described by Bassler *et al*. 1997, using 20 μl AI-2 extract and 180 μl 1:5000 diluted overnight cultured *V. harveyi *BB170 in AB medium [13]. Changes in bioluminescence upon addition of AI-2 were determined at 30°C every 30 min using a combined, automated luminometer-spectrometer (Anthos Labtech Lucy1). AI-2 activity was defined as the fold increase in light production in comparison with medium or buffer controls. For a single experiment, the *V. harveyi *bioassay was performed at least in duplicate for each sample. Experiments were repeated at least three times.

### RNA isolation and purification

Cultures were grown in triplicate as described above and bacteria were harvested during late logarithmic phase of growth (approximately 8 h, OD 0.3 and verified by viable counts) by centrifugation at 3000 *g *for 20 min. Pellets were resuspended in 1 ml of Tri Reagent (Sigma-Aldrich, UK) to which 0.2 ml chloroform (Sigma-Aldrich, UK) was added, mixed by vortexing and equilibrated at room temperature for 10 min. After centrifugation at 12000 *g *for 15 min the aqueous phase was removed and applied to Qiagen's RNeasy Mini columns for RNA purification according to the manufacturer's protocol. DNA removal was ensured by treatment with DNA-free (Ambion, UK) and the quality and quantity of RNA was checked using the Agilent 2100 Bioanalyzer (Agilent Technologies, UK).

### Construction of the *C. jejuni *DNA microarray

Internal DNA fragments corresponding to unique segments of the individual open reading frames (ORFs) in the annotated genome sequence of strain NCTC 11168 [45] were amplified by PCR using gene-specific primers (Sigma Genosys ORFmer set), then purified and spotted on GAPSII slides (Corning, USA) using an in-house Stanford designed microarrayer as previously described [46].

### Transcriptome analysis

Labelled cDNA was prepared from 15 μg RNA using Stratascript RT (Stratagene, UK) with the direct incorporation of Cy3 and Cy5 dyes (Amersham, UK), applied to microarrays, washed, scanned and statistically analysed as described by Holmes *et al*. [47]. Dye-swapping indicated that equal dye incorporation occurred. In short, duplicate microarray experiments were performed for each of the triplicate RNA samples and each ORF was present on the microarray in triplicate. The normalised data from each microarray were unified in one single dataset and reanalysed to identify the differentially expressed genes. Full methodology of the statistical analysis of the data was previously described [47].

### Production of AI-2 *in vitro*

AI-2 was synthesised essentially as described by Winzer *et al*., [26]. 2 mM *S*-adenosylhomocysteine (SAH, purchased from Sigma) in 10 mM sodium phosphate buffer, pH 7·7, was converted enzymatically to *S*-ribosylhomocysteine (SRH) through incubation with purified *E. coli *Pfs enzyme (100 μg ml^-1^) at 37°C for 1 h. Subsequently, purified *E. coli *LuxS (500 μg ml^-1^) was added, and the reaction mixture incubated for a further 2 h. SAH solutions were bubbled with helium before addition of the enzymes and the reaction mixtures were incubated in an anaerobic cabinet to prevent oxidation of the reaction products.

Levels of synthesised AI-2 were measured indirectly by quantification of homocysteine generated via the LuxS reaction. Homocysteine concentrations were determined using the Ellmans reagent as previously described [26]. AI-2 negative controls, for addition to control cultures, were prepared as follows: SRH was synthesised enzymatically as described above and adjusted to the concentration calculated for the AI-2 *in vitro *reaction, by dilution with reaction buffer and addition of homocysteine and adenine contained within the same buffer (also yielding the concentrations calculated to be present in the AI-2 *in vitro *reaction). Thus, apart from AI-2 (and other DPD derivatives) control and AI-2 positive sample had an identical composition.

### Production of AI-2 using crude cell-extracts

Cell pellets were harvested from exponentially growing *C. jejuni *cultures by centrifugation (3000 g for 20 min) and resuspended in an appropriate volume of 10 mM sodium phosphate buffer (pH 7.7) containing freshly added lysozyme (100 μg/ml; Sigma-Aldrich UK) and 'Bugbuster Benzonase' nuclease (1 μl ml^-1^; Novagen UK). After 30 min incubation at 37°C, debris was pelleted by centrifugation (10000 g for 15 min) and the crude cell extracts transferred to a new microfuge tube. To assess LuxS activity, cell-extracts were added in a 1:1 ratio to 4 mM SAH in sodium phosphate buffer, or to 2 mM SRH that was enzymatically produced from SAH as previously described [26]. In each case the resulting mixture was incubated for 2 hours at 37°C, mixed with an equal volume of chloroform, centrifuged, and the aqueous extract analysed for AI-2 activity using *V. harveyi *BB170 strain as a bioluminescent reporter [13]. As positive and negative controls for LuxS activity, cell extracts of *E. coli *MG1655 and DH5α, respectively, were used, as well as *C. jejuni *extracts incubated with buffer lacking the substrate.

### Addition of exogenous AI-2 to *C. jejuni *cultures

Cultures of *C. jejuni *NCTC 11168 and LuxS01 were grown as described above. After 2.5 h, *in vitro*-produced AI-2 was added to test cultures and the AI-2 negative mix was added to the control cultures as described above. This gave the cells time to reach exponential growth phase, and ensured AI-2 levels were maintained throughout the same growth period as is observed for the WT grown in MHB. Light assay samples were taken from controls and AI-2 samples immediately following addition of AI-2, then again at 8 h, before the cells were harvested and the RNA extracted for microarray expression analysis.

### Microarray Data

Microarray data is available on the Gene Expression Omnibus (GEO) database, http://www.ncbi.nlm.nih.gov/sites/entrez?db=gds. The accession number is GSE18455.

## Results

### *C. jejuni *produces AI-2 in MHB but not MEM-α

In line with observations made in other *C. jejuni *strains (NCTC 11168, 81116, and 81-176; [37,44,48], we found that in MHB, AI-2 production and motility by *C. jejuni *strain NCTC 11168 was abolished in an isogenic *luxS *mutant strain (LuxS01). We set out to understand the nature of the phenotypes reported for *C. jejuni luxS *mutants, which have been attributed to AI-2 mediated quorum sensing [44,48], or more recently at least in part to the role of LuxS in central metabolism [37]. To do this, we monitored the extracellular AI-2 profile during growth of *C. jejuni *NCTC 11168 and the isogenic *luxS *mutant strain (LuxS01) in a defined medium (MEM-α). As in the rich MHB media, disruption of *luxS *had no effect on growth in MEM-α (Data not shown). Interestingly, however, the growth medium had a marked effect on AI-2 production. For the first time we demonstrated that in MEM-α, AI-2 was hardly detectable (Data not shown). These data suggested that either AI-2 is not released from the cell in MEM-α, or that part of the AMC is not active under these conditions. To distinguish between the two possibilities, cell extracts of *C. jejuni *NCTC 11168 were prepared from cells harvested after 5 h growth and analysed for LuxS activity (see Methods for details). As positive and negative controls, cell extracts from *E. coli *strain MG1655 and strain DH5α containing a *luxS *frame shift mutation were used. Whole cell lysates were prepared and SRH added. Conversion to homocysteine and DPD were assessed using Ellmans reagent and the *V. harveyi *bioassay respectively. In agreement with previous studies [26,49] crude extracts of *E. coli *MG1655 contained detectable levels of homocysteine and DPD indicating LuxS activity (data not shown). However, neither compound was detectable in cell extracts of *E. coli *DH5α *luxS *mutant (negative control) or *C. jejuni *NCTC 11168. Neither growth in MHB nor MEM-α to the point when extracellular AI-2 levels are high in MHB (5 h) yielded *C. jejuni *NCTC 11168 extracts capable of converting SRH to homocysteine and DPD (i.e. exhibiting LuxS activity), suggesting either lack of DPD production (with detection limit for AI-2 of approx 6 μM) or rapid turnover.

### Mutation of *luxS *alters gene expression in a medium-dependent fashion

Microarrays were employed to compare the transcriptomes of *C. jejuni *wild type and *luxS *mutant grown in either MHB or MEM-α. This analysis, which was performed with cells harvested in late exponential growth (8 h after inoculation), revealed a number of differentially expressed genes [see Additional Files 1 and 2). Interestingly, most of the observed differences were media-dependent and associated with metabolic functions (i.e. catabolism, anabolism, transport, and energy production). There were also considerably more differentially expressed genes when the mutant and wild type strains were grown in MHB rather than in MEM-α (131 and 60 genes with a greater than twofold change respectively). 20 genes (comprising 14 probable transcription units) were differentially expressed in both media (thus comprising a third of the changes seen in MEM-α), suggesting that they were linked to loss of *luxS *function. These included genes with (putative) roles in amino acid and lactate uptake (Cj0982c and *lctP*, respectively), electron transport and respiration (Cj0037, Cj0073, Cj0074, Cj0075, *sdhC*) and oxidoreductase reactions (Cj1199, Cj0415). Some of the identified genes are known to play a role in anabolic pathways such as amino acid (e.g. *trpA*, *trpB*, *glnA*) and fatty acid (*fabI*) biosynthesis or central metabolism such as the tricarboxylic acid cycle (e.g. *sdhC*). Interestingly, gene Cj0982c has recently been shown to be involved in cysteine uptake. The upregulation of this gene in the *luxS *mutant is in agreement with the hypothesis that *luxS *mutants have an increased requirement for sulphur-containing amino acids [50]. In MEM-α, Cj0982 transcript levels were increased 7.2-fold. Together with Cj1199 (6.2-fold), Cj1200 (14.8-fold), and Cj1422c (9.1-fold) this was one of the most substantial changes observed under these conditions.

Interestingly, in MHB the largest changes in transcript abundance were observed for several putative stress response genes, which were all down-regulated in the *luxS *mutant. These include the putative *hrcA-grpE-dnaK *operon (Cj0757-Cj0758-Cj0759; 34.1, 28.7, and 21-fold changes, respectively), and a *clpB *chaperone homologue (Cj0509c; 28.1-fold). Smaller changes were also observed for the putative heat shock regulator *hspR *(Cj1230; 3.5-fold), *crpA *(Cj1229, encoding a *dnaJ *like protein; 4-fold) and the *groES-groEL *operon (Cj1220-Cj1221; 2.4 and 5.6-fold, respectively). Of these, only *clpB *transcript levels were also changed in MEM-α (2.4-fold). Transcript changes in MHB were also observed for the putative metabolic genes Cj1364 (*fumC*; 10.4-fold) and Cj0481 (a putative class I aldolase; 12.1-fold), as well as the conserved hypothetical Cj1631c (16.7-fold).

For the *C. jejuni luxS *mutant, reduced motility in MHB agar plates has been reported [35], a phenotype that was also confirmed in this study (data not shown). In agreement with these data, a set of 14 genes involved in flagella assembly and modification was found to be down-regulated in the MHB-grown *luxS *mutant. This included *flaA *(4.2 fold lower) reported previously to be reduced in a *luxS *mutant of strain 81116 [44]. Interestingly, the *luxS *mutant was also less motile in MEM-α based motility agar, although none of the flagellar genes differentially expressed in MHB were significantly altered. However in MEM-α the transcript levels of two different putative flagellar genes Cj0336c (*motB*) and Cj1312 were significantly reduced.

Two genes whose functions are associated with the AMC were found to be differentially regulated. In MHB, a 2.6-fold reduction of the *pfs *(Cj0117) transcript level was observed (Pfs is responsible for providing the LuxS substrate SRH), whereas in MEM-α the putative *metF *(Cj1202) gene was found to be down-regulated (2.4-fold).

### Transcriptional changes imposed by mutation of *luxS *are not caused by a lack of AI-2-dependent signalling

**Figure 1:**
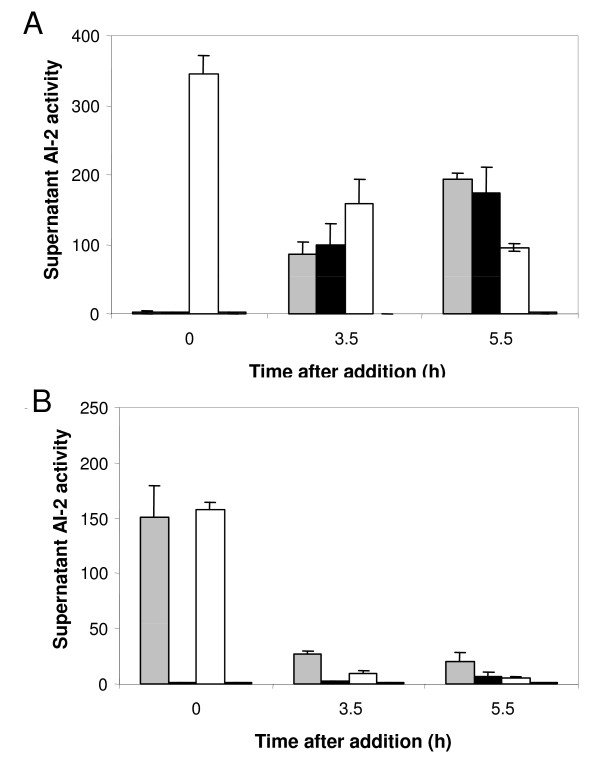
**Levels of exogenous AI-2 decrease during culture with *C.jejuni***. **Experiment A**: *In vitro *produced AI-2 (10 μM final concentration) was added to LuxS01 mutant after 2.5 h growth in MHB (white bar). A control buffer of enzymatically synthesised SRH supplemented with homocysteine and adenine control culture but lacking AI-2 was added to LuxS01 as a control (undetectable AI-2, at baseline). For comparison production of AI-2 by the wild type NCTC 11168 strain (grey bars) and also a replicate culture to which the control buffer was added (black bars) is shown. At 0, 3 and 5.5. h after addition of *in vitro *synthesized AI-2, its activity was measured in the culture supernatant using the *V. harveyi *light assay. The supernatant activity is expressed as the fold increase in light production relative to sterile medium as a control. **Experiment B**: results for a similar experiment to that described in experiment A, except that the cultures were grown in MEM-α. As AI-2 was not produced by *C. jejuni *in this medium it was added to both the LuxS01 mutant (white bars) and the wild type strain NCTC 11168 (grey bars) after 2.5 h in culture. As controls the buffer mixture lacking AI-2 was added to LuxS01 mutant (undetectable AI-2 thus not indicated) and the wild type strain (black bars). To investigate the response of LuxS01 and wild type strain to exogenously added AI-2, cells from experiments A and B were harvested in late exponential phase for RNA extraction and microarray gene expression analysis. In both experiments the error bars represent 1 SD from the mean.

To test the hypothesis that a lack of extracellular AI-2 was responsible for the observed changes in the LuxS01 transcriptome, *in vitro*-synthesized AI-2 was added to *C. jejuni *cultures. The amount of AI-2 added was adjusted so that the resulting AI-2 activity at the time point of cell harvest was comparable to that produced naturally by the wild type in MHB [see Figure 1]. In the case of the LuxS01 mutant, *in vitro *synthesized AI-2 was added to both MEM-α and MHB grown cultures after 2.5 h. As AI-2 was not produced by the parent strain in MEM-α, it was also added after 2.5 h to test whether gene expression would be affected by quorum signalling. Levels of AI-2 in the culture supernatant were measured immediately after addition (time 0) and then again after incubation for 3.5 h and 5.5 h. Interestingly, AI-2 activity diminished over time when added to the cultures of the *luxS *mutant grown in MHB or MEM-α [See Figure 1], suggesting that it was either inactivated or taken up by the cells. A similar reduction of AI-2 was observed for the WT grown in MEM-α. Despite this reduction, levels did not fall significantly below those in 3.5 h cultures where endogenous AI-2 was present. The cultures were harvested 5.5 h after AI-2 addition (i.e. 8 h of total growth) and RNA was extracted and assessed for transcriptional changes using DNA microarrays. No significant changes were observed between control cultures and those with AI-2 added in the *luxS *mutant. Parallel addition of exogenous AI-2 to the *luxS *mutant did not restore motility (see materials and methods, data not shown). This suggests that under the conditions of this study, extracellular AI-2 was not acting as a signal molecule and was not responsible for the transcriptome differences between wild type and *luxS *mutant.

## Discussion

### Differentially expressed genes in *C. jejuni *NCTC 11168 and its *luxS *mutant

In *Vibrio *spp, AI-2 functions as an extracellular signalling molecule. Many other bacteria also possess the enzyme LuxS and produce extracellular AI-2. Often, the phenotypic differences observed between *luxS *mutants and wild types have also been interpreted as AI-2 (i.e. quorum sensing)-dependent in these species. However, the observed phenotypic changes may be linked to a metabolic imbalance resulting from the disruption of AMC rather than the lack of an AI-2 signal [36,50]. Both underlying mechanisms have been presented as the basis for phenotypic modulation in *C. jejuni *[37,44,48]. In this study, the transcriptomes of exponentially growing *C. jejuni *NCTC 11168 and its *luxS *mutant were analysed using microarrays to distinguish between the two possibilities alongside examining potential strain-specific effects.

The transcriptomes were compared under a number of different conditions, which included growth in complex medium (MHB), in defined medium (MEM-α), and in the presence of *in vitro *synthesized AI-2. Since *C. jejuni *is asaccharolytic, the main carbon and energy sources drawn upon are likely to be amino acids such as serine, aspartate, glutamate and proline in both media [51-53]. 60 and 131 genes were differentially regulated when the strains were grown in MEM-α and MHB, respectively. Furthermore, 20 of these genes were differentially expressed in both media. Two of these genes (*cj1199 *and *cj1200*, located immediately downstream of *luxS*) were similarly modulated in the transcriptome analysis of the *C. jejuni *81-176 *luxS *mutant [37]. The difference in the MHB profiles generated by He *et al*., 2008 [37] and this study, may reflect an altered genetic background in the two strains or the different growth conditions (8 versus 17 hours of growth, late exponential versus stationary growth phase, and shaken versus static cultures). Comparing our data with that of He *et al*., 2008 [37], 14% of the genes showing differential expression in this study were also noted by He *et al*., 2008 [37] using microarrays and RT-PCR, with 60% of these being modulated in the same direction.

Overall, this indicates that inactivation of *luxS *influenced the expression of numerous genes, either directly or indirectly. However rather than a global affect on gene expression, there is a selection of genes modulated. None of these changes could be reversed by the addition of *in vitro *synthesized AI-2 under the conditions tested, suggesting that lack of AI-2 activity in the culture medium was not responsible for the observed differences. This contrasts to the situation in *Streptococcus mutans*, where exogenous AI-2 restored the level of gene expression some genes (e.g. acid tolerance, bacterocin synthesis and oxidative stress tolerance), but not others (including transcriptional regulators and membrane transporters)[54]. The exact mechanistic link between *luxS *mutation and the observed transcriptional changes is still not well understood. Several possibilities exist, which include an increased metabolic burden (due to the inability to salvage the homocysteine unit of SAH), accumulation of toxic intermediates, or a lack of DPD (which may be used as a precursor for biosynthetic purposes not connected with signalling). While the latter two possibilities can not be excluded, at least some of the observed changes are related to the metabolism of sulphur amino acids and, thus, can be interpreted as a response of the cell to the disruption of the AMC.

One of the genes up-regulated in the *luxS *mutant in both MHB and MEM-α is *cj0982c*, the product of which is a periplasmic binding protein specific for L-cysteine and has been proposed to be part of an ABC transporter involved in cysteine uptake [55]. The increased expression of this gene may reflect the need of the *luxS *mutant to counteract the loss of homocysteine salvage by increasing cysteine uptake from the environment. There is also some homology between Cj1200 and the D-methionine-binding lipoprotein MetQ involved in import of D-methionine, although there is a second closer homologue elsewhere on the *C. jejuni *chromosome.

Expression of the putative *metF *gene (Cj1202) was reduced in the *luxS *mutant grown in MEM-α and also in the stationary phase cells analysed by He *et al*., 2008 [37]. MetF (methylenetetrahydrofolate reductase) catalyses the formation of 5-methyltetrahydrofolate, a cofactor required for providing a methyl group during the conversion of homocysteine to methionine. The observed down-regulation of *metF *could be the consequence of a regulatory mechanism that responds to the reduced availability of homocysteine. A very similar situation is present in *S*. Typhimurium [20], where inactivation of *luxS *reduced the expression of *metE*. In *S*. Typhimurium, *metE *expression is positively regulated by MetR and homocysteine is known to considerably stimulate this activation [56,57]. Thus, reduced intracellular homocysteine level in the *S*. Typhimurium *luxS *mutant appears to be responsible for the reduced *metE *expression. A similar mechanism may have led to differential expression of *metF *in *C. jejuni *NCTC 11168 *luxS*. Another example of a differentially regulated AMC gene is *pfs *(Cj0117). This gene, which was up-regulated in the *luxS *mutant in MHB both in logarithmic phase (this study) and in stationary phase [37], is required for the conversion of SAH (for the purpose of detoxification and salvage of the resulting homocysteine and adenine moieties). The increase in *pfs *transcript levels could be the result of a regulatory mechanism responding to the concentration of AMC derivatives. Little is known about the regulation of *luxS *and *pfs *in different bacteria, but at least in *S*. Typhimurium, *pfs *expression depends on growth conditions and growth phase, whereas *luxS *is expressed constitutively [58].

The differential expression pattern of other genes is more difficult to explain and future work will need to address the regulatory mechanisms that respond to the intracellular changes associated with AMC disruption. Of particular interest in this context are the genes that encode putative homologues of enzymes involved in major metabolic pathways such as the tricarboxylic acid cycle (Cj0438 [*sdhB*], Cj0439 [*sdhC*], Cj1364 [*fumC*]), fatty acid biosynthesis (Cj0116 [*fabD*], Cj0441 [*acpP*], Cj1400 [*fabI*]), and amino acid biosynthesis (Cj0346 [*trpD*], Cj0347 [*trpF*], Cj0348 [*trpB*], Cj0349 [*trpA*], Cj0405 [*aroE*], Cj0891 [*serA*], Cj1599 [*hisB*], Cj1600 [*hisF*], Cj1601 [*hisA*]) only some of which are differentially expressed in both media [see Additional Files 1 and 2]. Also, it is not clear why major stress response genes were down regulated in the *luxS *mutant and why this change is only seen in MHB but not MEM-α, as a metabolic defect would have been expected to generate stress conditions, rather than to reduce them. It is also noteworthy that the profile of stress-response linked genes differentially expressed in this study was not the same as that observed in the MHB grown stationary phase cells analysed by He *et al*., 2008 [37], emphasizing that growth conditions have a significant influence upon gene expression. It is interesting that in this study the stress response was observed under the conditions where high levels of AI-2 were produced by the wild type. It must be emphasised, however, that these changes could not be reversed by the addition of exogenous AI-2, which argues against a role of quorum sensing in this response.

Contrary to a previous report [48], no downregulation of the cytolethal distending toxin genes (*cdtA*, *B *and *C*: *Cj0079c*, *Cj0078c*, *Cj0077c *respectively) was observed in the *luxS *mutant. This may be a reflection of the different growth times (we used 8 h, they 3 days), or strains used in the two studies (81116 by Jeon *et al*., 2005, NCTC 11168 here).

From Tables 1 and 2 [see Additional files 1 and 2] it is apparent that several sets of neighbouring genes were differentially regulated in a similar manner, suggesting that they may form operons and that their encoded proteins might function in the same pathways. For instance, the hypothetical iron-sulphur proteins Cj0073, Cj0074, Cj0075 appear to be transcriptionally linked with the putative lactate permease gene Cj0076 (*lctP*). Other examples include some of the flagellar genes, amino acid biosynthesis genes, and heat shock genes.

Of particular interest is the observed down-regulation of 14 putative flagella genes in the MHB-grown *C. jejuni *NCTC 11168 *luxS *mutant. This is in agreement with the reduction of motility in semi-solid MHB agar plates, as previously described for strains NCTC 11168 [35] and 81116 [44]. However, is in contrast to the recently published transcriptional data of the *luxS *mutant of *C. jejuni *strain 81-176 [37]. This may reflect the co-ordinate regulation exerted upon flagellar components and regulators, which, as He *et al*. 2008 [37] pointed out, is influenced by bacterial growth phase and environmental factors. Both genes encoding cheomotaxis proteins (Cj0363, Cj0284c (CheA) and Cj0144) as well as the flagellin genes *flaA *and *flaB *were among those found to be down-regulated in the present study. The former may impact upon motility [59], and the latter matches the findings of Jeon *et al*. (2003), who reported reduced *flaA *expression for *C. jejuni *81116 *luxS*, and showed that the flagellar structure was still preserved in this strain [44]. Reduced motility of the *C. jejuni *NCTC 11168 *luxS *mutant was also observed in MEM-α based agar plates (data not shown), although only the flagellar motor protein *motB *and the putative flagellar gene *cj1312 *were down-regulated under these conditions. A loss of LuxS function impacts on motility-associated genes in a range of different bacteria. For enterohemorrhagic *E. coli *(EHEC), *H. pylori*, and *C. jejuni *a role of AI-2 in the regulation of motility associated genes has been proposed [35,44,60,61]. At least for *C. jejuni*, this view is not supported by the data contained within the present study. The defect in motility caused by deletion of *luxS *in *H. pylori *was shown to be restored by addition of cell free medium containing AI-2 [62], but this could not be demonstrated for the *C. jejuni luxS *mutant in this study. The flagella regulator *flhA *was also shown to be induced by addition of AI-2 in a *luxS *mutant background providing further evidence for the role of AI-2 in the global regulation of flagella gene transcription [62]. In contrast, transcription of *flhA *was not altered in a *luxS *mutant of *C. jejuni *(this study and [37]). A phylogenetic tree of the LuxS protein revealed that the LuxS of *C. jejuni *is phylogenetically distant to that of *H. pylori *which could, in part, explain differences in function between the LuxS protein in *C. jejuni *and *H. pylori *[63]. Since it was probably acquired independently in the two species, the primary role taken on by *luxS *(gene regulation versus metabolic) would differ depending on what other pathways were already established.

### AI-2 production and degradation

Virtually no AI-2 activity was detectable when *C. jejuni *NCTC 11168 was grown in MEM-α. This could be due to a lack of AI-2 export, rapid intracellular turnover of DPD or AI-2 or lack of *luxS *or *pfs *expression and thus DPD synthesis. The latter possibility could not be ruled out, as it was not possible to detect Pfs and LuxS enzyme activity in cell extracts obtained from strain NCTC 11168 growing in MEM-α or in MHB. The reason for this remains unclear, as SAH and SRH conversion could be detected in similarly prepared *E. coli *cell extracts. It could be that in *C. jejuni*, enzyme activity levels are below those detectable in the assay. There is unlikely to be an absence of *pfs *expression in MEM-α, as previous studies have indicated modulated *pfs *expression [58] rather than an on/off control. Moreover, *pfs *mutations cause severe growth defects [64]. Given the absence of a growth defect in MEM-α, Pfs is likely to be present. In support of this, although the differential expression was not significant (confidence level was 18%, based on two separate P-values; slope and intercept), the *luxS *mutant had 1.9 fold more *pfs *expression than the WT in MEM-α. The overall differential gene expression detected in MEM-α suggests that the WT, but not the mutant produces LuxS.

Exogenous AI-2 activity gradually diminished when added to MHB or MEM-α grown *C. jejuni *cultures suggesting either uptake or degradation. However, *C. jejuni *does not seem to possess an AI-2 uptake system homologous to that found in *S*. Typhimurium and *E. coli*. In these organisms, AI-2 induces the expression of the *lsr *ABC transporter [16,20,22]. AI-2 is reported to be cleaved following phosphorylation into PG and another unidentified C3 fragment [65]. Modulation of the *lsr *operon (with approximately 10 fold magnitude) can be detected using microarrays to compare transcriptomes of WT and *luxS *mutants of *E. coli *[66] and although a similar system may exist in *C. jejuni*, the complete lack of AI-2-responsive genes suggests that uptake is not inducible by AI-2. He *et al*., 2008 [37] were also not able to select a potential uptake mechanism and noted the lack of sequence similarity that hampers the identification of ABC transporters involved in AI-2 uptake. Interestingly, extensive analysis could not identify an AI-2 receptor of either the ABC transporter or two component regulator type in *C. jejuni *[67]. Since the reported *E. coli lsr *regulation [66] was media-dependent, it cannot be ruled out that regulation of an uptake system in *C. jejuni *would occur under different conditions e.g. in biofilms [38]. Moreover, in addition to acting as a signal molecule under certain environmental conditions, the activity of AI-2 may be influenced by the phase of growth; for example, when extracellular AI-2 levels are maximal in late exponential/stationary phase. Further studies are therefore required to complete the characterization of the basis for phenotypic alterations caused by LuxS/AI-2 in *C. jejuni*, and these should carefully assess the effect of a range AI-2 concentrations and growth conditions to be fully conclusive.

## Conclusion

Whatever the *C. jejuni *strain investigated, it is apparent that mutation of *luxS *impacts upon expression of a subset of defined genes rather than with a pleotropic global change in the transcriptome. The genes modulated are primarily metabolic in nature and reflect the growth phase and nutritional environment of the cells analysed. Since exogenously added AI-2 had no impact on gene expression, it can be concluded that in *C. jejuni *strain NCTC 11168 this product of LuxS does not act as part of a quorum sensing machinery under the conditions used in this study.

## Authors' contributions

KH carried out the growth and phenotypic characterization of *C. jejuni*, the microarray analysis and drafted the manuscript. TT generated the AI-2 and performed its quantification. KW, JMW and KRH contributed to the design of the experiments and preparation of the manuscript. All authors read and approved the final manuscript.

## Supplementary Material

Additional file 1 **Table Comparing relative transcript levels in NCTC 11168 and LuxS01 grown in MHB**. Table showing relative transcript levels of genes differentially expressed in LuxS01 compared to *C. jejuni *NCTC11168 in MHB. Click here for file

Additional file 2 **Table Comparing relative transcript levels in NCTC 11168 and LuxS01 grown in MEM-α**. Table showing relative transcript levels of genes differentially expressed in LuxS01 compared to *C. jejuni *NCTC11168 in MEM-α. Click here for file
