# Supplementary material for: AI-2 does not function as a quorum sensing molecule in Campylobacter jejuni during exponential growth in vitro
Source: BMC Microbiol. 2009 Oct 8;9:214. doi: 10.1186/1471-2180-9-214 (PMC2772989; doi:10.1186/1471-2180-9-214)
Supplement: Additional file 1 — Table Comparing relative transcript levels in NCTC 11168 and LuxS01 grown in MHB. Table showing relative transcript levels of genes differentially expressed in LuxS01 compared to C. jejuni NCTC11168 in MHB. [file 1471-2180-9-214-S1.doc]

**Table 1: Comparison of relative transcript levels in NCTC 11168 and LuxS01 grown** **in MHB.** Wild type *C. jejuni* NCTC 11168 and its isogenic *luxS* mutant (LuxS01) were grown in MHB as described in Fig 1 [see Additional file 1]. Comparative RNA expression profiling was performed with cells harvested after 8 h using DNA microarrays and analysed as described in Methods. Genes highlighted in bold were also differentially expressed in the strains when grown in MEM-α medium [see Additional file 3]. Genes associated with * were also differentially regulated in the study of He *et al*., 2008 [37].

| **Gene No.**  **(gene name)** | Fold Change | **Description of Proposed Function** |
| --- | --- | --- |
| **Genes with altered transcript levels in LuxS01 compared to NCTC 11168** | | |
| Flagellar genes | |  |
| *cj0041** (*fliK*) | - 2.3 | Putative flagellar hook-length control protein |
| *cj0042**(*flgD*) | - 2.4 | Putative flagellar hook assembly protein |
| *cj0526c* (*fliE*) | - 2.6 | Putative flagellar hook-basal body complex |
| *cj0547*(*flaG*) | - 6.2 | Possible flagellar protein |
| *cj0548** (*fliD*) | - 5.2 | Putative flagella cap protein |
| *cj0549** (*fliS*) | - 3.2 | Putative flagellin specific chaperone |
| *cj0687c** (*flgH*) | - 2.9 | Putative flagella L-ring protein precursor |
| *cj0697** (*flgG2*) | - 2.5 | Putative flagellar basal-body rod protein |
| *cj0720c* (*flaC*) | - 4.4 | Flagellin |
| *cj0887c** (*flaD*) | - 2.9 | Putative flagellin and/or related hook-associated protein |
| *cj1331** (*ptmB*) | - 3.2 | Acylneuraminate cytidyltransferase (flagellin modification) |
| *cj1332** (*ptmA*) | - 2.9 | Putative oxidoreductase (flagellin modification) |
| *cj1338c** (*flaB*) | - 3.9 | Flagellin |
| *cj1339c* *(*flaA*) | - 4.2 | Flagellin |
| *cj1462** (*flgI*) | - 2.9 | Flagellar P-ring protein |
|  |  |  |
| Electron transport | |  |
| ***cj0037c*** | **- 5.9** | **Putative cytochrome C** |
| ***cj0074c*** | **- 4.2** | **Putative iron-sulfur protein** |
| ***cj0075c*** | **- 3.8** | **Putative oxidoreductase iron-sulfur protein** |
|  |  |  |
| Chemotaxis proteins | |  |
| *cj0262c* | - 2.7 | Putative methyl-accepting chemotaxis signal transduction protein |
| *cj0284c* (*cheA*) | - 3.8 | Chemotaxis histidine kinase |
| *cj0144* | - 2.9 | Methyl-accepting chemotaxis signal transduction protein |
|  |  |  |
| Heat shock proteins | |  |
| *cj0757* (*hrcA*) | - 34.1 | Putative heat shock regulator |
| *cj0758* (*grpE*), *cj0759** (*dnaK*) | - 28.7, - 21 |  |
| *cj1220** (*groES*), *cj1221** (*groEL*) | - 2.4, - 5.6 | 10kDa and 60kDa heat shock chaperonin |
| *cj1230* (*hspR*) | - 3.5 | Putative heat shock transcriptional regulator |
| *cj1229* (*cbpA*) | - 4 | Putative curved-DNA binding protein DnaJ class heat shock chaperonine |
| *cj1230* (*hspR*) | - 3.5 | Putative heat shock transcriptional regulator |
|  |  |  |
| Transport/binding proteins | |  |
| *cj0182* | 2.7 | Transmembrane transport protein |
| *cj0412* | 3.0 | Putative ATP/GTP binding protein |
| ***cj0982c*** | **5.5** | **amino-acid transporter periplasmic binding protein specific for cysteine** |
| ***cj0076c* (*lctP*)** | **- 4.0** | **L-lactate permease** |
| *cj0484* | - 4.6 | Transmembrane transport protein |
| *cj1231* (*kefB*) | - 3.6 | Putative glutathione-regulated potassium-efflux system protein |
| *cj1450* | - 6.3 | Putative ATP/GTP-binding protein |
| *cj1584c* | - 2.9 | Putative peptide ABC-transport system periplasmic peptide-binding protein |
| *cj1630* (*tonB2*) | - 11.3 | Putative tonB transport protein |
| *cj1655c** (*nhaA1*) | - 2.4 | Na(+)/H(+) antiporter |
| *cj1656c** | - 3.7 | Hypothetical protein component of ABC transporter |
| *cj1659** (*p19*) | 2.6 | Putative integral membrane protein p19, ABC transporter permease |
|  |  |  |
| Other unknown/putative functions | |  |
| *cj1077, cj1219c, cj1235,* *cj1380* | 3.8, 2.5, 2.3,  2.3 | Putative periplasmic proteins |
| *cj0089, cj1678* | 2.9, 3.4 | Possible lipoproteins |
| *cj0598* | 2.7 | Putative membrane protein |
| *cj0118, cj0119, cj0949c,* *cj1325, cj1368* | 2.4, 2.6, 2.3,  4.5, 2.4 | Hypothetical proteins |
| *cj0058, cj0770c, cj0771c, cj0772c, cj0854c,* ***cj0864,*** *cj0876c* | - 3.3, - 2.8, - 3.5,  - 3.4, - 3.5, - **3.6**,  - 2.5 | Putative periplasmic proteins |
| *cj0455c, cj0553, cj0987c, cj0989, cj1468* | - 4.1, - 2.6, - 7.8,  - 3, 3 | Putative membrane proteins |
| *cj0044c cj0055c, cj0056c,* ***cj0073c****,* ***cj0170****, cj0391c, cj0416, cj0644, cj0719c, cj0739, cj0760, cj0761, cj0977, cj1316c, cj1463, cj1465, cj1631c, cj1671c* | - 3.5, - 3.8,- 3.0,  - **4.6,** - **2.6**, - 2.9,  - 7.4, - 2.8, - 2.7,  - 2.9, - 8.2, - 3.0,  - 3.1, - 3.7, - 2.8,  - 3.5, - 16.7, | Hypothetical proteins |
|  |  |  |
| Regulatory functions | |  |
| *cj0890c* | 2.3 | Putative sensory transduction transcriptional regulator |
| *cj1189c* | - 3.1 | Putative signal transduction sensor protein |
| *cj1506c* | - 2.8 | Putative MCP-type signal transduction protein |
|  |  |  |
| General metabolic functions |  |  |
| *cj0068* (*pspA*) | 2.7 | Protease |
| *cj0088* (*dcuA*) | 2.4 | Putative anaerobic C4-dicarboxylate transporter |
| ***cj0348* (*trpB*)** | **2.5** | **Tryptophan synthase beta chain** |
| ***cj0349* (*trpA*)** | **2.9** | **Tryptophan synthase alpha chain** |
| *cj0405* (*aroE*) | 3 | Shikimate 5-dehydrogenase |
| *cj0438** (*sdhB*), ***cj0439***  **(*sdhC*)** | 2.7, **3.2** | Putative succinate dehydrogenase iron-sulfur subunit B and **subunit C** |
| *cj0503c* (*hemH*) | 2.8 | Putative ferrochelatase |
| *cj0516* (*plsC*) | 2.6 | Putative 1-acyl-SN-glycerol-3-phosphate acyltransferase |
| *cj0585* (*folP*) | 2.4 | Putative dihydropteroate synthase |
| *cj0637c* (*mrsA*) | 2.4 | Putative peptide methionine sulfoxide reductase |
| *cj0827* (*truA*) | 3.4 | Putative tRNA pseudouridine |
| *cj0843c* | 2.4 | Putative secreted transglycosylase |
| *cj0891c* (*serA*) | 2.3 | D-3-phosphoglycerate dehydrogenase |
| *cj0117** (*pfs*) | 2.6 | 5’-methylthioadenosine\S-adenosylhomocysteine nucleosidase, AI-2 biosynthesis/SAM metabolism |
| *cj1319* | 2.3 | Putative nucleotide sugar dehydratase |
| ***cj1399c* (*hydA2*)** | **2.4** | **Putative Ni/Fe-hydrogenase small subunit** |
| ***cj1400c* (*fabI*)** | **3** | **Putative enoyl-(acyl-carrier-protein) reductase (NADH)** |
| *cj1481c* | 2.7 | Putative helicase |
| *cj1599* (*hisB*) | 2.9 | Imidazoleglycerol-phosphate dehydratase/histidinol-phosphatase |
| *cj1600* (*hisH*) | 2.8 | Amidotransferase |
| *cj1601* (*hisA*) | 2.8 | Phosphoribosylformimino-5-aminoimidazole |
| *cj1645* (tkt) | 2.4 | Transketolase |
| *cj0045c* | - 4.7 | Putative iron-binding protein |
| *cj0441* (*acpP*) | - 2.5 | Acyl carrier protein |
| *cj0481* | - 12.1 | Putative class I aldolases, Dihydrodipicolinate synthase subfamily |
| *cj0482* (*uxaA*’), *cj0483*  (*uxaA’*) | -4.3, - 7.0 | Putative altronate hydrolase N-terminus and C-terminus |
| *cj0688* (*pta*) | - 2.4 | Putative phosphate acyltransferase |
| ***cj0699c* (*glnA*)** | **- 4.5** | **Glutamine synthetase** |
| *cj0817* (*glnH*) | - 2.6 | Glutamine-binding periplasmic protein |
| *cj1192* (*dctA*) | - 3.1 | Putative C4-dicarboxylate transport protein |
| ***cj1199**** | **- 2.6** | **Putative iron/ascorbate-dependent oxidoreductase,** AI-2 biosynthesis/SAM metabolism |
| ***cj1200**** | - **5.8** | Putative periplasmic protein, AI-2 biosynthesis/SAM metabolism |
| *cj1293* | - 2.5 | Possible sugar nucleotide epimerase/dehydratae |
| *cj1315c* | - 2.9 | Amidotransferase |
| ***cj1425c*** | **- 3.3** | **Putative sugar kinase** |
| *cj1537c* (*acs*) | - 3.1 | Acetyl coenzyme A synthetase |
| *cj1566c* (*nuoN*), | - 3.7 | NADH dehydrogenase I chain N |
|  |  |  |
| Miscellaneous |  |  |
| ***cj0415*** | **- 2.6** | **Putative oxidoreductase subunit** |
| ***cj0479* (*rpoC*)** | **- 2.5** | **DNA-directed RNA polymerase beta chain** |
| *cj0485* | - 2.6 | Putative oxidoreductase |
| ***cj0509c* (*clpB*)** | **- 28.1** | **ATP-dependent CLP protease ATP-binding subunit** |
| *cj1237c* | - 3.5 | Possible phosphatase |
| *cj1364* (*fumC*) | - 10.4 | Fumarate hydratase |
