# Supplementary material for: AI-2 does not function as a quorum sensing molecule in Campylobacter jejuni during exponential growth in vitro
Source: BMC Microbiol. 2009 Oct 8;9:214. doi: 10.1186/1471-2180-9-214 (PMC2772989; doi:10.1186/1471-2180-9-214)
Supplement: Additional file 2 — Table Comparing relative transcript levels in NCTC 11168 and LuxS01 grown in MEM-α. Table showing relative transcript levels of genes differentially expressed in LuxS01 compared to C. jejuni NCTC11168 in MEM-α. [file 1471-2180-9-214-S2.doc]

**Table 2: Comparison of relative transcript levels in NCTC 11168 and LuxS01 grown in MEM-α**

Wild type *C. jejuni* NCTC 11168 and its isogenic *luxS* mutant (LuxS01) were grown in MEM-α as described in Fig 1 [see Additional file 1]. Comparative RNA expression profiling was performed with cells harvested after 8 h using DNA microarrays and analysed as described in Methods. Genes highlighted in bold were also differentially expressed in the strains when grown in MHB [see Additional file 2]. Genes associated with * were also differentially regulated in the study of He *et al*., 2008 [37].

| **Gene No.**  **(gene name)** | **Fold Change** | | **Description of Proposed Function** |
| --- | --- | --- | --- |
| **Genes with altered transcript levels in LuxS01 compared to NCTC 11168** | | | |
| Flagellar proteins | |  | |
| *cj0336c* (*motB*) | - 4.4 | Putative flagellar motor protein | |
| *cj1312* | - 2.2 | Possible flagellar protein | |
|  |  |  | |
| Electron transport | |  | |
| ***cj0037c*** | **- 4.0** | **Putative cytochrome C** | |
| ***cj0074c*** | **- 3.8** | **Putative iron-sulfur protein** | |
| ***cj0075c*** | **- 4.2** | **Putative oxidoreductase iron-sulfur protein** | |
|  |  |  | |
| Transport/binding proteins |  |  | |
| ***cj0076c* (*lctP*)** | **- 3.5** | **L-lactate permease** | |
| *cj1161c* | - 2.9 | Putative cation-transporting ATPase | |
|  |  |  | |
| Unknown/putative functions | |  | |
| *cj0375* | 2.2 | Putative lipoprotein | |
| *c0965c, cj1710c* | 2.6, 2.4 | Hypothetical proteins | |
| ***cj0982c*** | **7.2** | **amino-acid transporter periplasmic binding protein specific for cysteine** | |
| *cj0365c* | - 2.4 | Putative outer membrane channel protein | |
| *cj0561c,* ***cj0864****,*  *cj1256c, cj1725* | - 2.8, - **3.3**,  - 3.3, - 2.4 | Putative periplasmic proteins | |
| *cj1003c* | - 3.1 | Putative integral membrane proteins | |
| *cj0522* | - 2.3 | Putative membrane proteins | |
| ***cj0073c****,* ***cj0170****, cj0494, cj1162, cj1242* | **- 4.8**, - **2.6**, - 2.8,  - 2.9, - 2.4 | Hypothetical proteins | |
| *cj1057c* | - 2.5 | Putative coiled-coil protein | |
| *cj1084c* | - 3.3 | Putative ATP/GTP-binding protein | |
|  |  |  | |
| Regulatory functions | |  | |
| *cj1492c* | 3.2 | Putative two-component sensor | |
|  |  |  | |
| General metabolic functions | |  | |
| *cj0116* (*fabD*) | 2.4 | Malonyl CoA-acyl carrier protein transacylase | |
| *cj0346** (*trpD*) | 2.2 | Anthranalite synthase component 11 | |
| *cj0347* (*trpF*) | 3 | N-(5’-phosphoribosyl) anthranilate isomerase | |
| ***cj0348* (*trpB*)** | **2.6** | **Tryptophan synthase beta chain** | |
| ***cj0349* (*trpA*)** | **2.3** | **Tryptophan synthase alpha chain** | |
| ***cj0439* (*sdhC*)** | **2.6** | **Putative succinate dehydrogenase subunit C** | |
| *cj0538* (*oorC*) | 2.2 | OORC subunit of 2-oxoglutarate:acceptor oxidoreductase | |
| *cj0581* | 2.2 | Putative NTPase | |
| *cj1317* (*neuB3*) | 2.2 | N-acetylneuraminic acid synthetase | |
| ***cj1399c* (*hydA2*)** | **3.6** | **Putative Ni/Fe hydrogenase small subunit** | |
| ***cj1400c* (*fabI*)** | **3.2** | **Putative enoyl-(acyl-carrier-protein) reductase** | |
| *cj0011c* | - 3.4 | Putative non-specific DNA binding protein | |
| *cj0308c* (*bioD*) | - 2.2 | Putative dethiobiotin synthetase | |
| ***cj0699c*** | **- 3.9** | **Glutamine synthetase** | |
| *cj0723c* | - 3.0 | Putative integral membrane zinc-metalloprotease | |
| *cj0798c* (*ddlA*) | - 2.2 | Putative D-alanine—D-alanine ligase | |
| *cj0865* (*dsbB*) | - 3.0 | Putative disulfide oxidoreductase | |
| *cj0917c* (*cstA*) | - 3.2 | Carbon starvation protein A homolog | |
| ***cj1199**** | **- 6.2** | **Putative iron/ascorbate-dependent oxidoreductase,** AI-2 biosynthesis/SAM metabolism | |
| ***cj1200**** | - **14.8**, | Putative periplasmic proteins, AI-2 biosynthesis/SAM metabolism | |
| *cj1202** (*metF*) | - 2.4 | 5,10-methylenetetrahydrofolate reductase | |
| *cj1344c* | - 2.4 | Putative glycoprotease | |
| *cj1421c, cj1422c* | - 3.2, - 9.1 | Possible sugar transferases | |
| ***cj1425c*** | **- 2.1** | **Putative sugar kinase** | |
| *cj1385* (*katA*) | - 4.0 | Catalase | |
|  |  |  | |
| Miscellaneous |  |  | |
| *cj0414,* ***cj0415*** | - 2.4, -**3.0** | Putative oxidoreductase subunits | |
| ***cj0479* (*rpoC*)** | **- 2.4** | DNA-directed RNA polymerase beta chain | |
| ***cj0509c* (*clpB*)** | **- 2.4** | **ATP-dependent CLP protease ATP-binding subunit** | |
|  |  |  | |
| Ribosomal proteins |  |  | |
| *cj0961c* (*rpmH*) | - 2.9 | 50S ribosomal protein L34 | |
| *cj1691c* (*rplR*) | - 2.2 | 50S ribosomal protein L18 | |
| *cj1694c* (*rpsN*) | - 2.2 | 30S ribosomal protein S14 | |
| *cj1699c* (*rpmC*) | - 2.6 | 50S ribosomal protein L29 | |
|  |  |  | |
